# The association between healthy eating index score with semen parameters in infertile men: A cross-sectional study

**DOI:** 10.18502/ijrm.v20i11.12360

**Published:** 2022-12-10

**Authors:** Kimia Leilami, Azadeh Zareie, Mehran Nouri, Milad Bagheri, Mahsa Shirani

**Affiliations:** ^1^Nutrition Research Center, Shiraz University of Medical Sciences, Shiraz, Iran.; ^2^Students' Research Committee, Isfahan University of Medical Sciences, Isfahan, Iran.; ^3^Department of Community Nutrition, School of Nutrition and Food Science, Food Security Research Center, Isfahan University of Medical Sciences, Isfahan, Iran.; ^4^Students' Research Committee, School of Nutrition and Food Science, Shiraz University of Medical Sciences, Shiraz, Iran.; ^5^Department of Community Nutrition, School of Nutrition and Food Science, Shiraz University of Medical Sciences, Shiraz, Iran.; ^6^Health Policy Research Center, Institute of Health, Shiraz University of Medical Sciences, Shiraz, Iran.

**Keywords:** Diet, Healthy, Semen analysis, Infertility, Cross-sectional studies.

## Abstract

**Background:**

Infertility has been a major problem for young couples in recent years. One way to assay the diet quality is the healthy eating index (HEI), related to infertility.

**Objective:**

This study aims to assess the association between the HEI score with semen parameters in Iranian infertile men.

**Materials and Methods:**

Two hundred and sixty eligible men (18-55 yr), were referred to the major infertility clinic in the summer of 2018 and entered this cross-sectional study. Based on the 5 ${}^{\text{th}}$ edition of the world health organization laboratory manual, semen parameters including sperm concentration, volume, motility, and morphology were analyzed, and to specify the dietary intake of individuals a 168-item questionnaire was used. Also, to calculate the total HEI score, all 13 components based on HEI-2015 components and scoring standards were summed up.

**Results:**

Participants in the highest tertile, had no difference in mean sperm parameters with those in the lowest tertile in the crude model. No significant association was found between sperm parameters and HEI score tertiles in the crude model, even after adjustment for potential confounders, except for concentration (OR: 0.39 and CI: 0.15, 0.99, p = 0.04). Participants in the highest tertile had a lower risk of abnormal concentration and motility in the crude model. The risk of abnormal concentration decreased, and motility increased in the adjusted model.

**Conclusion:**

In this cross-sectional study, there was no significant relationship between HEI and sperm indexes, except for sperm concentration. Therefore, more studies need to be done in the future.

## 1. Introduction

Among couples who plan to conceive, one in 6 fail to do so after 12 months of regular unprotected sexual intercourse, known as infertility (1). In Iran, the infertility rate is above the global average (a quarter of couples vs. approximately 15% of couples globally) (2). About 25% of the causes of infertility are related to men, including decreased sperm concentration and quality (3). Currently, the etiology of suboptimal semen quality is unknown (4). Some studies suggest that many physiological, environmental, and genetic factors, including oxidative stress and diet, can play a role (5). Some modifiable lifestyle factors, such as diet and physical activity can be of clinical and public health importance (1).

There is a relationship between some components of the diet and serum reproductive hormones that may affect modulating spermatogenesis, sperm maturation, and fertilizing ability (6). One study showed the pattern in reproductive hormones was different between the body mass index (BMI) groups. The decreased semen quality was observed in men with BMI < 20 kg/m^2^ or BMI > 25 kg/m^2^, although the mechanisms may not be the same between them (7). Some studies showed a negative correlation between BMI and sperm concentration or total sperm count (8, 9).

One way to assay diet quality is the healthy eating index (HEI). HEI is defined as “a measure for assessing whether a set of foods aligns with the Dietary Guidelines for Americans”. The HEI score indicates overall dietary quality and separate component scores, which can be inspected collectively to reveal a pattern of quality regarding multiple dietary dimensions (10). Some studies have shown an association between HEI and chronic diseases such as obesity (11), diabetes (12), cardiovascular disease, and cancer (13), but there was not enough research on men's infertility. Diets that are high in carbohydrates, fiber, folate, vitamin C, lycopene, fruits, and vegetables have been associated with higher sperm quality (14). Conversely, a low-quality diet consisting of a high-calorie diet, trans-fatty acids, saturated fats, cholesterol, added sugars, and alcohol have been associated with detrimental effects on fertility (5, 14). Hence the rate of HEI may be effective on infertility.

Previous studies have focused on the effect of individual nutrients on men's infertility; however, there is a lack of evidence about the effect of dietary patterns on sperm parameters. Therefore, this study aimed to assess the association between the HEI score with semen parameters in Iranian infertile males.

## 2. Materials and Methods

### Participants

Two hundred and seventy infertile men, who referred to infertility center of Isfahan province, entered to this study. The study was conducted in Isfahan, Iran in the summer of 2018. Participants had a history of primary or secondary infertility and were aged between 18-55 yr-old. Men with a history of urinary infection, genital disease, and metabolic disease such as diabetes, cardiovascular disease, cancer, osteoporosis or renal disease and also use of cytotoxic drugs, anticoagulants and supplements, were not included in the study (15). Finally, 254 men were included in the analysis. (The detailed account of methods and data collection have been previously published) (16).

### Assessment of semen parameters

According to the 5 ${}^{\text{th}}$ edition of the World Health Organization (WHO) laboratory manual (15), the processing and analysis of 4 dependent semen parameters, including concentration, total motility, normal morphology, and semen volume, were done (17).

### Assessment of dietary intakes

The dietary intakes were assessed by a validated 168-item food frequency questionnaire (FFQ). The FFQ validity and reproducibility for use in Iranian adults have been previously shown (18). Nutritionist IV software (N4) modified for Iranian foods was utilized to extract dietary intakes based on FFQ. (More detailed dietary intakes assessment in detail are provided in a previous published article) (19).

### HEI

Calculation of the HEI scores included 13 components: “(1) whole fruit; (2) total fruit; (3) greens and beans; (4) total vegetables; (5) total protein foods; (6) seafood and plant proteins; (7) whole grains; (8) dairy; (9) fatty acids; (10) refined grains; (11) sodium; (12) added sugars; and (13) saturated fats”. The maximum point allocated to each of the first 6 groups is 5 points, and for the other groups, it is 10 points.

The highest consumption of the first 9 components, scored 0-5 or 10, for example, the lowest consumption score was 0 and the highest consumption score was 5 or 10. The score of other 4 components (including total fat, saturated fatty acids, cholesterol, and sodium) reversely was calculated. To calculate the total HEI score, all 13 components according to HEI-2015 components were summed up together.

### Assessment of other variables

Demographic data, medical history, alcohol and tobacco use, and supplements intake were collected by a well-trained nutritionist and a structured questionnaire in face-to-face interviews. Bodyweight in kilograms (kg) and height (cm) were measured by standard procedures. BMI as the ratio between weight in kilograms and height in meters squared (kg/m^2^) was calculated.

### Ethical considerations

All participants signed the consent form and voluntarily entered the study. The study was ethically approved by Ethical Committee of Isfahan University of Medical Sciences, Isfahan, Iran (Code: IR.MUI.RESEARCH.REC.1397.232).

### Statistical analysis

In this study, participants were categorized based on tertiles of the HEI. The Kolmogorov-Smirnov test was used to access the normality of the data. For comparing the continuous and categorical variables across tertiles of HEI, a one-way analysis of variance (ANOVA) and Chi-square test were used. Two different multivariable logistic regressions were used to assess the relation between HEI and odds of abnormal sperm parameters (crude and adjusted model).

Age and energy intake were controlled in the first adjusted model, whereas BMI, marriage time, educational status, physical activity, alcohol, and smoking history were added to the second.

In this study, like as the previous study by Shirani et al. (16), semen quality parameters were grouped based on WHO cut points. “For example, sperm concentration less than 15 M/ml is considered abnormal, while a concentration more than 15 M/ml is considered normal. Participants with all tested semen parameters statistical analyses were carried out using SPSS for Windows software (version 20.0), SPSS Inc, and Chicago IL. P-value < 0.05 was considered statistically significant”. (The detailed account of methods and data collection have been previously published) (16).

## 3. Results

In table I, baseline characteristics of participants are shown based on tertile of HEI score. BMI and waist circumference were high in the first tertile of the HEI score. There was a significant change between tertiles of HEI with BMI. Participants in the highest tertile of HEI score had no difference in mean sperm parameters compared to those in the lowest tertile (p > 0.05).

In table II, the energy-adjusted dietary nutrients and food items intakes of participants, are shown through tertiles of HEI score. In the last tertile of HEI, participants had a higher intake of carbohydrate, protein, fat, fiber, monounsaturated fatty acid, vitamin B ${}_{6}$ , B ${}_{9}$ , C, magnesium, zinc, iron, total fruits, total vegetables, legumes and nuts, and total protein foods, but lower intake of energy, saturated fatty acid, refined grains, and added sugars compared to lowest tertile.

Intakes of polyunsaturated fatty acid, cholesterol, vitamin A, B ${}_{12}$ , calcium, selenium, and dairy did not differ in HEI score tertiles. multivariable-adjusted odds ratio (OR) and 95% confidence intervals (CIs) for abnormal sperm parameters across tertiles of HEI are shown in table III. The results in this table are based on ordinal logistic regression and the first tertile was considered as the reference group. Sperm parameters were grouped according to WHO cut points to normal and abnormal semen parameters.

The association between the last tertile of HEI score with sperm concentration was not significant in the crude and first adjusted model (OR: 0.55 and CI: 0.28, 1.08, p = 0.08 and OR: 0.52 and CI: 0.26, 1.05, p = 0.07), but a protective association was seen in the full adjusted model (OR: 0.39 and CI: 0.15, 0.99, p = 0.04), which means that participants in the highest tertile of HEI score had 61% lower risk for abnormal sperm concentration than those in the last tertile.

**Table 1:** Basic characteristics of participants across the tertiles of HEI scores


**Variable**		**HEI**			
		**T1 (n = 90)**	**T2 (n = 88)**	**T3 (n = 76)**	**P-value**
Age (yr)*		31.46 ± 4.62	30.88 ± 3.90	31.13 ± 3.89	0.66
Body mass index (kg/m^2^)*		26.36 ± 3.84	26.58 ± 3.89	28.08 ± 4.50	0.01
Waist (cm)*		93.45 ± 9.49	94.01 ± 10.19	96.94 ± 11.28	0.07
Marriage time (yr)*		5.25 ± 2.929	5.36 ± 2.99	5.96 ± 3.23	0.28
Physical activity (Met.h/day)*		29.20 ± 1.99	29.50 ± 2.21	29.07 ± 2.17	0.54
**Volume (ml)**		4.14 ± 0.22	3.99 ± 0.22	4.39 ± 0.23	0.47
**Concentration ( × 10^6^/ml)**		11.01 ± 1.65	13.25 ± 1.67	15.10 ± 1.80	0.24
**Total motility (%)**		30.03 ± 1.90	26.73 ± 1.92	31.78 ± 2.07	0.19
**Normal morphology (%)**		5.01 ± 1.06	3.27 ± 1.07	3.81 ± 1.16	0.50
Smoking history**					
	**Yes**	35 (38.9)	32 (36.4)	27 (35.5)	
	**No**	55 (61.1)	56 (63.6)	49 (64.5)	0.89
Alcohol history**					
	**Yes**	14 (15.6)	18 (20.5)	21 (27.6)	
	**No**	76 (84.4)	70 (79.5)	55 (72.4)	0.16
Supplement use**					
	**Yes**	29 (32.2)	22 (25.0)	29 (38.2)	
	**No**	61 (67.8)	66 (75.0)	47 (61.8)	0.19
Education status**					
	**Less than high school**	12 (13.3)	25 (28.4)	19 (25.0)	
	**High school diploma**	36 (40.0)	19 (21.6)	23 (30.3)	0.03
	**Bachelor degree or higher**	42 (46.7)	44 (50.0)	34 (44.7)	
*Data presented as Mean ± SD (one-way ANOVA). **Data presented as n (%) (Chi-square test). HEI: Healthy eating index, T1: First tertile, T2: Second tertile, T3: Third tertile					

**Table 2:** Dietary intakes of participants across the tertiles of HEI scores


**Variable**		**HEI**			
		**T1 (n = 90)**	**T2 (n = 88)**	**T3 (n = 76)**	**P-value**
**Nutrient items**					
	**Energy (kcal/d)**	2437.77 ± 74.68	2410.33 ± 78.55	2410.33 ± 64.95	0.003
	**Carbohydrate (g/d)**	342.24 ± 11.15	344.51 ± 11.03	402.68 ± 11.02	0.02
	**Protein (g/d)**	91.03 ± 2.86	87.52 ± 2.56	106.62 ± 3.06	0.001
	**Fat (g/d)**	86.31 ± 5.32	84.98 ± 4.62	90.33 ± 3.70	0.002
	**Dietary fiber (g/d)**	36.90 ± 1.85	39.06 ± 1.44	46.88 ± 1.93	0.01
	**SFA (g/d)**	28.89 ± 1.19	26.22 ± 1.34	28.02 ± 1.13	< 0.001
	**MUFA (g/d)**	27.73 ± 1.13	28.15 ± 1.65	29.63 ± 1.33	0.02
	**PUFA (g/d)**	15.53 ± 0.68	17.21 ± 1.05	18.38 ± 0.92	0.08
	**Cholesterol (mg/d)**	328.68 ± 23.25	280.17 ± 20.25	340.71 ± 23.53	0.25
	**Vitamin A (RAE/d)**	792.16 ± 44.36	738.21 ± 37.65	904.43 ± 50.12	0.40
	**Vitamin E (mg/d)**	12.70 ± 0.56	13.82 ± 0.88	14.24 ± 0.67	0.14
	**Vitamin B_6_ (mg/d)**	2.07 ± 0.06	2.03 ± 0.05	2.51 ± 0.06	< 0.001
	**Vitamin B_9_ (µg/d)**	533.74 ± 16.06	527.14 ± 12.49	616.48 ± 17.93	0.02
	**Vitamin B_12_ (µg/d)**	6.23 ± 0.37	5.60 ± 0.33	6.60 ± 0.36	0.44
	**Vitamin C (mg/d)**	210.65 ± 13.01	229.92 ± 12.06	295.61 ± 14.98	0.003
	**Calcium (mg/d)**	1166.07 ± 48.01	1144.88 ± 40.76	1359.83 ± 47.63	0.24
	**Magnesium (mg/d)**	423.41 ± 15.79	416.59 ± 13.05	528.67 ± 14.77	< 0.001
	**Selenium (mg/d)**	113.35 ± 5.50	106.30 ± 3.41	129.87 ± 5.42	0.18
	**Zinc (mg/d)**	13.95 ± 0.48	12.95 ± 0.44	16.15 ± 0.51	0.003
	**Iron (mg/d)**	17.01 ± 0.60	16.77 ± 0.50	20.09 ± 0.60	0.02
**Foods items (g/d)**					
	**Whole grains **	29.98 ± 3.55	59.49 ± 8.78	96.01 ± 10.25	< 0.001
	**Refined grains **	335.09 ± 15.63	292.66 ± 11.21	259.88 ± 15.96	0.001
	**Total fruits **	454.40 ± 21.04	563.07 ± 41.08	595.27 ± 21.94	0.002
	**Total vegetables **	411.73 ± 20.43	416.15 ± 17.91	503.20 ± 20.59	0.001
	**Legumes **	28.41 ± 2.12	34.46 ± 1.91	36.87 ± 2.56	0.01
	**Nuts **	14.88 ± 1.34	16.37 ± 1.52	22.05 ± 2.64	0.01
	**Dairy **	428.71 ± 25.41	464.22 ± 20.95	501.74 ± 26.22	0.10
	**Total protein foods **	169.31 ± 6.86	172.75 ± 7.02	198.17 ± 8.86	0.01
	**Added sugars **	124.27 ± 10.47	93.54 ± 5.91	52.82 ± 4.57	< 0.001
Data presented as Mean ± SE. ANOVA test. HEI: Healthy eating index, T1: First tertile, T2: Second tertile, T3: Third tertile, SFA: Saturated fatty acid, MUFA: Monounsaturated fatty acid, PUFA: Polyunsaturated fatty acid					

**Table 3:** Crude and multivariable-adjusted odds ratios and 95% CIs for abnormal sperm parameters across tertiles of the HEI scores


**Variable**		**HEI**			
		**T1 (n = 90)**	**T2 (n = 88)**	**T3 (n = 76)**	**P-value**
**Volume (≤ 2 ml)**					
	**Model I ${}^{\text{a}}$ **	1.00	1.42 (0.74-2.70)	1.34 (0.69-2.63)	0.36
	**Model II ${}^{\text{b}}$ **	1.00	1.41 (0.74-2.70)	1.49 (0.75-2.97)	0.23
	**Model III ${}^{\text{c}}$ **	1.00	1.26 (0.53-3.00)	1.17 (0.47-2.86)	0.74
**Concentration (≤ 15 M/ml)**					
	**Model I ${}^{\text{a}}$ **	1.00	0.97 (0.48-1.94)	0.55 (0.28-1.08)	0.08
	**Model II ${}^{\text{b}}$ **	1.00	0.99 (0.49-1.99)	0.52 (0.26-1.05)	0.07
	**Model III ${}^{\text{c}}$ **	1.00	0.93 (0.35-2.45)	0.39 (0.15-0.99)	0.04
**Total motility (40 ≤ %)**					
	**Model I ${}^{\text{a}}$ **	1.00	1.23 (0.62-2.44)	0.89 (0.45-1.76)	0.77
	**Model II ${}^{\text{b}}$ **	1.00	1.22 (0.61-2.43)	0.93 (0.46-1.87)	0.88
	**Model III ${}^{\text{c}}$ **	1.00	1.30 (0.48- 3.49)	1.03 (0.39-2.73)	0.96
**Normal morphology (≤ 4%)**					
	**Model I ${}^{\text{a}}$ **	1.00	1.4 (0.42-4.58)	0.83 (0.27-2.48)	0.75
	**Model II ${}^{\text{b}}$ **	1.00	1.36 (0.41-4.52)	0.66 (0.21-2.10)	0.54
	**Model III ${}^{\text{c}}$ **	1.00	1.31 (0.19-8.83)	0.49 (0.08-2.79)	0.45
Data presented as an odds ratio (95% CIs). Logistic regression, a: crude. b: adjusted for age and energy intake. c: additionally adjusted for BMI, marriage time, educational status, physical activity, alcohol, and smoking history. HEI: Healthy eating index, T1: First tertile, T2: Second tertile, T3: Third tertile					

## 4. Discussion

The study was carried out on 254 infertile men, in general, no significant relationship was found between HEI and sperm indexes, except in concentration. The energy-adjusted dietary nutrients and food intakes of participants through tertiles of HEI score showed that participants in the last tertile of HEI had a higher intake of carbohydrate, protein, fat, fiber, monounsaturated fatty acid, vitamin B_6_, B_9_, C, magnesium, zinc, iron, total fruits, total vegetables, legumes and nuts, and total protein foods, but lower intake of energy, saturated fatty acid, refined grains, and added sugars compared to lowest tertile. These data indicate that the HEI score can be related to more variation in diet and dietary intakes of vitamins and minerals increased in the last tertile of the HEI score. Although, intakes of polyunsaturated fatty acid, cholesterol, vitamin A, B_12_, calcium, selenium, and dairy did not differ in HEI score tertiles.

The results of the study, which was carried out on 280 men in Israel, demonstrated that participants with more HEI scores had higher concentration and sperm motility. Also, men who were in the last quartiles of the Adjusted Healthy Eating Index (AHEI) were better in terms of all concentration, total count, morphology, and sperm motility; therefore, this study recommended that following AHEI dietary principles can increase the quality of semen in men (20). Such differences in other food patterns have also been reported, for example, the results of one study showed the effect of Mediterranean food patterns on sperm indexes such as total sperm count (21). In contrast, the results of a study on 225 men referring to the fertility clinic in Greece showed that those with a lower Mediterranean score were 2.6 times more likely to have abnormal sperm concentration, total sperm count, and motility (22). Most of the previous investigations have evaluated the association of single food with the quality of sperm. In fact, a limited number of studies applied food patterns (23, 24), and further studies are needed to be done in the future.

Studies showing the positive impact of healthy dietary patterns on the quality of sperm are increasing (5), and the potential mechanism usually involves preventing chronic diseases (25, 26). For example, the study by van Bussel et al. declared that higher AHEI scores could reduce cardiovascular disease, diabetes, and cancer by decreasing the concentration of inflammatory factors and endothelial dysfunction (27). Also, in healthy food patterns, lower consumption of some foods such as trans-fatty acids, processed meat, refined cereals, and sweetened beverages (21, 28) and more intake of fruits and vegetables that are a source of various vitamins and minerals (29, 30), can have a positive effect on the quality of sperm. A recent meta-analysis study concluded that adherence to a healthy dietary pattern compared to an unhealthy dietary pattern can significantly improve seminal quality (31). But on the other hand, none of the AHEI, Mediterranean, and dietary approaches to stop hypertension diets affect the concentration of hormones (20). It seems that the effect on the level of reproductive hormones through the diet is very complex, because a cross-sectional study on 215 healthy young men, identified that not only Mediterranean food pattern but also Western food pattern, has failed to make significant changes in the level of hormones (21).

Another finding in this cross-sectional study was that participants who had a higher score of HEI had more BMI than those with a lower score, which was expected because of more intake of 3 macronutrients, carbohydrates, proteins, and fats. In another study, there was a reverse relationship between the total number of sperm and anthropometric indexes (p = 0.02), and motility of sperm has a negative relationship with BMI (p = 0.026) and waist circumference (p = 0.049) (32). Obesity and overweight in men can affect both the molecular and physical structure of sperm and disrupt their reproductive ability (33). Therefore, anthropometric indexes can also be an important and effective factor in the quality of sperm and are better to be measured than dietary patterns.

The limitation of this study, such as other cross-sectional studies, is the inability to prove the causal relationship. Although actions such as access to enough sample size, considering inclusion and exclusion criteria, and adjustment for age, energy intake, BMI, physical activity, marriage time, educational status, smoking, and alcohol history were made for the reliability of results, but there is a possibility that remains confounded.

To evaluate the food intake last year, we used a validated FFQ in the Iranian population that was completed by a trained person as an interview. However, this method is dependent on the memory of participants. For evaluating the quality of sperm, we used semen sampling according to WHO recommendations; most of the evidence reported that only one semen sample is sufficient for epidemiological studies (34, 35). The most important point of the current study was that instead of investigating the impact of a single food or a group of food, we evaluated food patterns and their relationship with the quality of sperm, which was rarely done in previous studies (36).

## 5. Conclusion

In this cross-sectional study, there was no significant relationship between HEI and sperm indexes, except for sperm concentration. Therefore, more studies need to be done in future.

##  Conflict of Interest

The authors have no conflict of interest.
